# Barriers and facilitators to accessing and engaging with arts-based non-pharmacological interventions for people living with dementia: A systematic review

**DOI:** 10.1177/14713012251333017

**Published:** 2025-04-12

**Authors:** Megan Polden, Megan Rose Readman, Tahlia Barnard, Abigail Godfrey, Annabel Gray, Clarissa Giebel

**Affiliations:** Department of Primary Care & Mental Health, 4591 University of Liverpool, UK; NIHR Applied Research Collaboration North West Coast, UK; Department of Health Research, Lancaster University, UK; Department of Primary Care & Mental Health, 4591 University of Liverpool, UK; NIHR Applied Research Collaboration North West Coast, UK; Department of Psychology, 151567 Lancaster University, UK; Department of Primary Care & Mental Health, 4591 University of Liverpool, UK; Department of Primary Care & Mental Health, 4591 University of Liverpool, UK; NIHR Applied Research Collaboration North West Coast, UK

**Keywords:** dementia, arts interventions, barriers, facilitators, access, engagement

## Abstract

**Background and Aims:**

Non-pharmacological arts interventions are increasingly being recognised as effective and beneficial ways to support and improve cognition and general well-being for people with dementia. However, accessing and engaging with beneficial arts interventions and support services can be challenging for people with dementia and their carers and it is important to understand barriers and facilitators that may impede access. This systematic review aimed to synthesise evidence on the barriers and facilitators to accessing and engaging with arts interventions and services for people living with dementia.

**Methods:**

We systematically searched five electronic databases (PubMed, PsycINFO, CINAHL, Scopus, Web of Science) for studies reporting barriers and facilitators to accessing and engaging with arts interventions for people with dementia in July 2024, screening a total of 7815 articles. Nineteen papers were deemed eligible for inclusion in this review including 567 people with dementia, 320 unpaid carers and 355 paid carers.

**Results:**

This review highlights key facilitators and barriers to accessing and engaging with arts interventions for people with dementia. Key facilitators included the assistance of volunteers, the inclusion of unpaid carers in the sessions, and the effective facilitation of sessions. Barriers to access and engagement were identified for people with dementia and their unpaid carers such as increased reliance on unpaid carers and a lack of training and time for paid carers.

**Conclusions:**

Increased awareness of these barriers and facilitators could aid in designing future arts interventions and support services to promote wider access and engagement for people with dementia and their carers.

## Introduction

Non-pharmacological arts interventions are becoming increasingly recognised as important and beneficial for people living with dementia, with growing research highlighting benefits to both cognition (Sharew, 2022) and general well-being (Meyer & O’Keefe, 2020). Approximately 55 million people are living with dementia across the world (World Health Organisation, 2023) with more than 980,000 living with dementia in the UK (Alzheimer’s Society, 2021). Dementia is a neurodegenerative condition that results in a decline in cognitive functioning including memory and language (Smits et al., 2015) and impacts a person’s ability to perform everyday activities such as preparing a hot meal or getting dressed (Andersen et al., 2004; Giebel et al., 2017). In addition to deterioration in cognitive functioning, dementia often leads to behavioural and psychological changes such as agitation, depression, hallucinations and reductions in inhibitory control at times leading to culturally inappropriate behaviours (Calsolaro et al., 2021; McKeith & Cummings, 2005; Schwertner et al., 2022). Although some pharmacological interventions can help with the management of cognitive symptoms of dementia (Watts et al., 2023), they often have limited effectiveness for behavioural symptoms (Gerlach & Kales, 2020). There are also often negative side effects that accompany medications to relieve behavioural symptoms which may worsen quality of life (Maidment et al., 2018). Due to this, there is a need for effective non-pharmacological interventions to reduce and aid in the management of behavioural symptoms of dementia (O’Donnell et al., 2022).

Arts interventions for people with dementia use creative activities such as music, visual arts (drawing and painting) or storytelling to support cognitive, emotional and social well-being (Newman et al., 2019; Young et al., 2016). These activities are thought to evoke memories and positive feelings when memory or verbal communication may have deteriorated (Ehresman, 2014). Arts interventions have proven beneficial to people with dementia, improving quality of life and cognition and reducing depressive symptoms (Bourne et al., 2021; Letrondo et al., 2023; Ward et al., 2021). Indeed, a vast amount of research has demonstrated how these art activities can improve well-being by promoting positive feelings, reducing anxiety, fostering social intervention and engagement with carers and other members of the community (Jeppson et al., 2022; Lee et al., 2022a; Windle et al., 2018). For example, music therapy has been effective as familiar songs can activate memory recall and stimulate conversation, helping individuals feel more connected to their identities and past experiences (El Haj et al., 2012; Irish et al., 2006; Juslin & Västfjäll, 2008). Similarly, visual arts activities, like painting, allows people with dementia to express themselves and engage in sensory-rich experiences, which can be calming, rewarding and boost confidence (Barroso et al., 2022; Lea & Synnes, 2021; Sauer et al., 2016). In community and healthcare settings, arts interventions are recommended to be integrated into dementia care programs (NICE, 2018) to create a more holistic and person-centred approach, focusing not only on symptom management but creating a stimulating and engaging environment for people living with dementia. These activities provide meaningful stimulation, encourage creative expression, and promote social interaction, all of which can improve the well-being of people with dementia and can additionally support carers well-being (Bourne et al., 2021).

However, delivering sustainable, affordable and accessible post-diagnostic support for people with dementia is a challenge globally (Alzheimer’s Disease International, 2016) with evidence suggesting a current lack of services and post-diagnostic support that is appropriate and accessible (Alzheimer Europe, 2020; Cooper et al., 2016). People with dementia often struggle to get the support and care they need after a diagnosis and people often encounter multiple barriers to accessing support services (Caprioli et al., 2023; Giebel et al., 2023). In addition to these barriers, several inequalities impact support and care access including living situation (living alone), age, gender, geographical location, education, dementia type and household finances (Giebel, 2024). To tackle and reduce these barriers and inequalities for people with dementia it is important to understand what barriers are present to accessing and engaging with support services and interventions that research has shown to be effective methods for improving quality of life and wellbeing.

Despite these clear benefits, many people with dementia do not have access to arts interventions to support their well-being. A lack of access may be the result of various factors such as a lack of available services in their area, travel or increased reliance on carers (Armstrong et al., 2022). Although research has assessed barriers to post-diagnostic support more generally, systematic reviews to date have not examined and synthesised the evidence base on specific barriers that may prevent access and engagement to arts interventions. Understanding the facilitators and barriers faced by people with dementia when trying to access arts-based interventions specifically could aid in making support services more accessible and promote engagement. To date, systematic reviews examining arts-based interventions for people with dementia have largely focused on the impacts of these services rather than their accessibility and barriers to access. With a clear literature basis for the benefits of arts-based interventions, it is important to understand the barriers and facilitators in relation to the accessibility of these services. Therefore, this systematic review aims to synthesise current literature on barriers and facilitators to accessing and engaging with art-based interventions for people with dementia.

## Methods

For this review, a narrative synthesis approach (Popay et al., 2006) was used, and the PRISMA guidelines were followed (Moher et al., 2009). The review was registered on PROSPERO (CRD42024557540) prior to the review commencing.

### Eligibility

Studies were eligible for inclusion if participants with dementia had received a formal diagnosis, and they had taken part in an arts intervention where barriers and facilitators to access or engagement were assessed. Studies that included another clinical group (i.e., Parkinson’s disease) were still eligible for inclusion but only the data from the dementia group was used in this review. Studies were also eligible for inclusion if they had data from carers of people with dementia on the barriers and facilitators to accessing and engaging with art interventions. Qualitative, quantitative (primary or secondary data analysis; surveys; electronic health record data), and mixed-methods studies were eligible for inclusion in this review. The studies must have examined barriers and facilitators to accessing or engaging with a non-pharmacological art intervention for people with dementia. All types of arts interventions were eligible for inclusion including but not exclusive to music-based interventions, drama interventions, painting and drawing interventions. Arts interventions that were only delivered in an online format were not eligible for inclusion. Studies were limited to primary data only and publications written in English. There were no restrictions on publication dates or country. The main outcome measure was establishing barriers and facilitators experienced by people with dementia when accessing and engaging with arts-based non-pharmacological interventions.

### Search strategy

Five databases (PubMed, PsycINFO, CINAHL, Scopus, Web of Science) were searched on 08/07/2024. The search string included the population of interest (people with dementia), arts interventions (music, painting, drama etc) and also terms relating to access or inequalities including any relevant dictionary terms across our included databases, as well as free-text search terms identified from relevant articles (see supplemental materials for full search teams). Searches were restricted to articles published in English there were no restrictions on publication dates. Thesis and conference abstracts were excluded. Open Science Framework (OSF), Medrxiv, and Social Science Research Network (SSRN) pre-print archives were searched for unpublished studies. The online program Rayyan was used to manage search outputs and screen articles (Ouzzani et al., 2016). After the removal of duplicates, four researchers (MP, TB, AG, AG) completed title and abstract screening ensuring each article had been processed independently by at least two researchers, and two researchers (MP and TB) completed the full-text screening independently. One researcher (MP) searched the OSF pre-print archive and SSRN pre-print servers and a second researcher (TB) independently checked for eligibility for inclusion.

### Data selection and extraction

Data were extracted independently by two researchers (MP and TB) from all articles that met the inclusion criteria. The following information was extracted: bibliographic information, the country the study was conducted in, participant sample and characteristics (e.g., gender, age, dementia severity), intervention delivery (e.g., type of intervention, setting, number of sessions), study design and information on barriers and facilitators identified in the study.

### Risk of bias

The risk of bias was assessed using the Critical Appraisal Skills Programme (CASP) checklist for qualitative research (CASP, 2024). The CASP tool examines factors such as whether the research aims were clear, methodology is appropriate and whether it addresses the aims of the study. The CASP examined 10 criteria and provides an indication of the quality of the research. For the CASP a scoring system is not assigned however it is advised that if you are unable to answer ‘yes’ to the first 2/3 questions then the evidence is likely to be of poor quality. The risk of bias assessments were completed independently by two researchers (MP and TB) and any differences were resolved through discussion.

## Results

### Study selection

**Figure 1.**
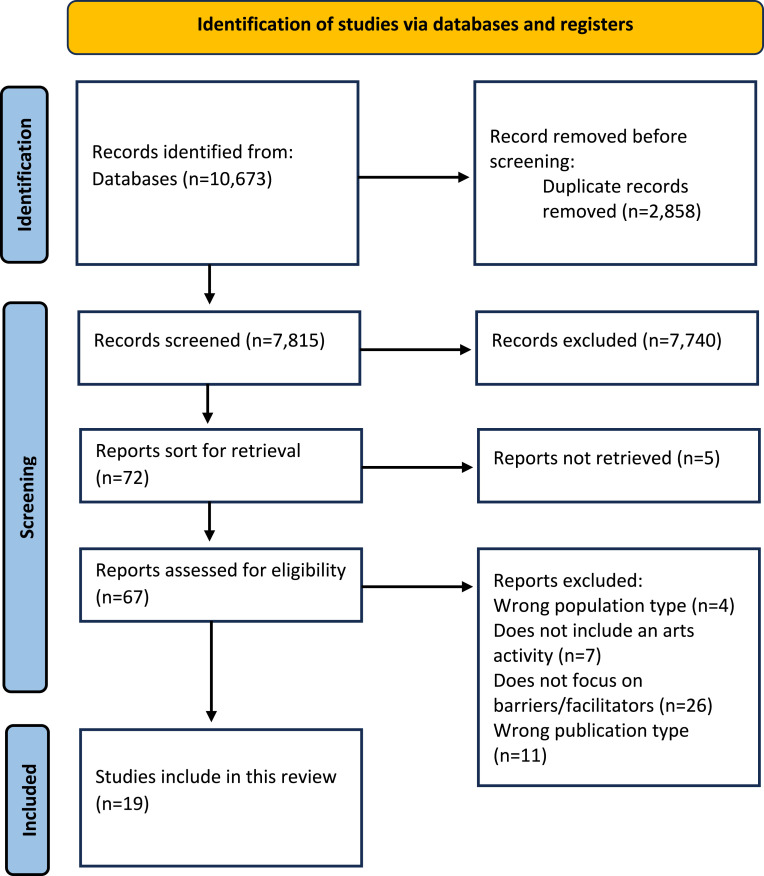
PRISMA flowchart.

The initial search identified 10,673 records and after the removal of duplicates, 7815 articles were screened during stage 1 (title and abstract screening). Following this, 103 articles were included for stage 2 full-text screening, resulting in 19 articles eligible for inclusion in this review (See Figure 1).

### Characteristics of included studies

The 19 studies included in this review originated from Australia, Canada, Germany, Ireland, South Africa, Taiwan, the UK and the US (see Table 1). Sixteen studies focused on music with six focusing on music listening, six group singing, three on music making and music therapy and one on dance. A further two studies focused on museum interventions and two others included a mix of arts activities, such as painting, pottery, music and drawing. The majority of the studies focus on art activities that were delivered in a care home setting (*n* = 10) with eight delivered in a community setting and one delivered in both residential care homes and at home.

**Table 1.** Overview of included studies.

Author (Year)	Country	Population and sample size	Type of arts intervention	Setting of intervention	Barriers identified	Facilitators identified
Bazooband et al. (2023)	Australia	12 people with dementia 3 females, 6 males, 3 with young onset dementia	Mix arts interventions including singing, pottery, drawing, painting.	Community based	Finding out information about the availability of programmes.	Factors such as location, timing, suitable venues and transport were important for participants. Participants felt less anxious going to interventions when supported by someone that they knew.
					Limited knowledge about existing arts activities in the community.	
					Reliance on spouses or carers to find information.	
Clark et al. (2018)	Australia	12 people with dementia and family care giver dyads	20 therapeutic group singing sessions	Community based	The organisation, engagement and enthusiasm demonstrated by the facilitators.	Concerns about future sustainability of the groups.
					An accepting environment.	
Clark et al. (2021)	Australia	20 participants: 8 spousal dyads, 2 family dyads	Group therapeutic songwriting and music making	Community-based		Accessibility of activity.
						Music appreciation that extended beyond sessions.
Dahms and Haesner (2018)	Germany	40 participant dyads: People with dementia and their relatives	Music listening	Implementing music at home and in care homes	Reliance on carers	Living in a care home or attending day activities to have more access to musical activities.
					Needing support to play live music or handle instruments.	
Dimopoulos-Bick et al. (2019)	Australia	21 staff participated in focus groups. 153 surveys completed by 49 people with dementia, 69 family members and 35 staff	Music listening with personalised playlists	Rural healthcare settings	Availability of staff, staff time and training.	Integration of protocols for selection of music and development of playlists with care practices.
					Funds for accreditation fee.	High level of staff engagement.
					Equipment storage, distribution, hygiene and safety.	Help from volunteers.
Inoue et al. (2024)	US	261 people with dementia with 148 in the intervention group and 113 in the control group.	Music listening with personalised playlists	Care homes	A lack of staff engagement, music management, and a lack of resident engagement.	Device maintenance and accessibility
Lee et al. (2022c)	Australia	24 participants: 4 people with dementia, 5 family members of people with dementia, 15 care home staff	Group music therapy and recreational choir singing	Residential care homes		Accessibility of activity and ease of participation.
						Flexible and person-centred approach used by session facilitators.
						Use of familiar songs and providing lyrics.
Lee et al. (2022b)	Ireland	7 participants: 3 people with early-stage dementia and 4 family carers	Group singing intervention	Community centre		Medium of music, active engagement, making valuable contributions, changing relationship dynamics.
Livingston et al. (2016)	US	>300 participants: People with dementia and carers	Museum gallery tour and 60–90-minute art-making activity	Museum galleries and learning centre		Environmental adaptations such as activities held at less busy and noisy times.
						Skilled facilitators specialising in working with people with dementia.
Mangione (2013)	US	35 participants: 13 museum personnel, 5 outside personnel working with educators, 7 carers, 5 carer/person with dementia dyads	Art museum gallery tour	Contemporary art museum		Help form volunteers, adapting to peoples needs, inclusive and openminded approach to artwork interpretation.
McDermott et al. (2014)	UK	53 participants: 12 care home residents with dementia, 4 day hospital clients with dementia, 15 family carers, 14 care home staff, 8 music therapists	Music playing and listening	Residential care home	Difficulty meeting people’s individual music tastes, staff being familiar with residents music taste, finding time and space for music activities, perceived limited interest in therapy from front-line staff.	Music as an accessible and flexible activity,
Petts and Urmston (2022)	UK	3 family carers	Dance	Community dance class		Availability and access, social engagement, improved relationship connectivity between carer and person with dementia, form of caregiving respite.
Sixsmith et al. (2010)	UK	10 people with dementia	Music listening	Residential care home	Difficulty using the music player without help, difficulty expressing desire to listen to music and choice of music.	Accessible activity, easy to use and implement in many different settings such as social, solitary, at home, public.
Stuart-Röhm et al. (2023)	South Africa	20 participants: 10 carers, 10 people with dementia	Carer singing	Residential care home	A lack of dementia specific training, not having music preferences in care plans, carers assumption that singing would upset residents or be time consuming, initial discomfort of carers singing in front of colleagues.	
Stuart‐Röhm et al. (2024)	South Africa	39 formal carers	Carer singing	Residential care home	Residents not always engaging and responding to singing, not knowing familiar songs to residents, challenges in adapting routine when resident resisted participation.	
Sung et al. (2011)	Taiwan	214 nursing staff caring for people with dementia in long-term facilities	Use of music in care homes	Residential care home	Lack of knowledge, training, skills, regarding music therapy, lack of resources and time, lack of confidence to give music therapy.	
Thompson et al. (2023)	Australia	14 participants: 7 people with dementia, 7 care partners	Choir singing	Remini sing choirs- community based	Hesitancy to try something new, singing anxiety.	Attending with carers facilitated accessibility, welcoming environment, location, venue, regularity and timings.
Unadkat et al. (2017)	UK	34 participants: 17 people with dementia and their 17 carers	Group singing	Community-based singing groups		Accessible and enjoyable activity, effective facilitation, active participation, participants in a group setting.
Yous et al. (2024)	Canada	37 participants: 16 family carers, 21 care home staff	Namaste care including massages, aromatherapy, music.	Residential care home	Dependency on information from families, finding space for activities, staff shortages, obtaining necessary supplies, retention of volunteers.	Help from volunteers to reduce staff burden.

Studies that reported on dementia type (*n* = 3) included people with various dementia subtypes such as Alzheimer’s Disease, vascular dementia, mixed dementia and other dementia types, although the majority of studies did not report on dementia type (*n* = 16). Across the 19 studies, 567 people with dementia were included, 320 unpaid carers and 355 paid carers were included in this review.

### Facilitators to accessing and engaging with in-person arts interventions

A number of studies found that including unpaid carers in arts interventions facilitated access and engagement (Clark et al., 2021; Petts & Urmston, 2022; Thompson et al., 2023). Petts and Urmston (2022) examined community-based dance classes for people with dementia and their carers and found that the inclusion of carers within the dance class facilitated access. The group was designed to meet the needs of both carers and people with dementia, and it was highlighted that this provided caregiving respite as an enjoyable shared experience. This sentiment was echoed by Clark et al. (2021) with family members appreciating the opportunity to be involved in therapeutic songwriting sessions with the person with dementia they cared for and stating that the sessions were made more enjoyable when attended together. Similarly, Thompson et al. (2023) found that including unpaid carers within interventions was practical and convenient and that participating together improved relationships between the person with dementia and their carer. Similar to this, a facilitator to accessing arts interventions was living in nursing homes or going to a daily care institution as this provided access to music through participating in musical activities and also through the continuous playing of the radio in nursing homes (Dahm’s & Haesner, 2018).

Linked to this, some studies indicated that certain arts activities may facilitate access due to the nature and accessibility of the activity itself. The accessibility of music as a form of arts activity and intervention was discussed in several studies, particularly in relation to the accessible nature for people with varying stages of dementia and musical ability (Lee et al., 2022b, 2022c; McDermott et al., 2014; Sixsmith et al., 2010). Sixsmith et al. (2010) developed a music player for people with dementia and found that music is widely accessible for people of all abilities and that music playing is transferable to many different settings and situations such as social, solitary, at home, and in public. McDermott et al. (2014) further highlighted the accessibility of many music interventions for people with dementia by finding that listening to music or playing musical instruments was stimulating even for residents who often appeared disengaged. The flexibility of music interventions allowed for different levels of participation and multiple ways for people with dementia to interact with the music, such as through foot tapping, clapping, singing or dancing. Additionally, Clark et al. (2021) found that group therapeutic songwriting was described as more accessible than recreational music experiences that had become inaccessible for people with dementia. For participants who hadn’t previously shown interest in music, therapeutic songwriting was described as breaking a barrier to music engagement and giving them a new appreciation for music which transferred beyond the sessions.

Evidence also highlighted that making mindful accommodations to arts activities can facilitate access. Livingston et al. (2016) examined the impact of a museum visit and art-making activities and found that making environmental accommodations such as hosting the event during less busy and noisy times and providing listening devices to help people hear and reduce surrounding distractions improved accessibility and engagement. Studies also highlighted that having art therapists who specialised in working with people with dementia can facilitate access and engagement. Similar themes were found by Mangione (2013) when examining access to an art museum intervention, finding that having volunteers on hand to assist with the tours and educators that can adapt to the needs of the group facilitated access and engagement. Bazooband et al. (2023) also found that making environmental accommodations such as wayfinding and receiving support while attending the activity improved accessibility and engagement. Wayfinding refers to the process of navigating and orienting oneself within an environment often with the support of environmental cues such as maps, landmarks and spatial design (Jamshidi et al., 2020). Strategies can be implemented to support people with cognitive impairments navigate a space more effectively and with greater ease improving accessibility. Findings suggested that adequate information, easy access, a welcoming and inclusive atmosphere, effective facilitators, and a judgement-free environment are desirable features which are congruent with dementia-inclusive arts activities.

Staff training to effectively deliver and facilitate arts interventions was highlighted as a facilitator (Clark et al., 2018; Lee et al., 2022c; Unadkat et al., 2017). Dimopoulos-Bick et al. (2019) reported that engagement facilitators included the availability, format and timeliness of the staff training and the implementation of clear protocols for delivering the intervention within existing clinical practice. Additionally, Unadkat et al. (2017) found that the accessibility of singing, combined with effective facilitation, created an environment for active participation and enjoyment. The group effect mediated further benefits for the person with dementia and their carers, when combined, this increased benefits for the couple through participation in new experiences. Lee et al. (2022c) further highlighted the importance of skilled facilitation with Clark et al. (2018) suggesting that participants valued the support provided by the two facilitators who were enthusiastic, organised, communicated well and provided take-home singing activities to support engagement between sessions. These studies highlight that effective and timely staff training and effective facilitators improve access and engagement with music activities and interventions.

### Barriers to accessing and engaging with in-person arts interventions

Despite some studies suggesting that the inclusion of unpaid carers facilitated access and engagement (Clark et al., 2021; Petts & Urmston, 2022; Thompson et al., 2023), in some studies, reliance on carers was viewed as a barrier to accessing certain art interventions. In Dahms and Haesner (2018), the authors reported that people with dementia living at home relied on carers to help facilitate access to music within the home, such as switching on the radio or another music system. People with dementia also struggled to play live music or handle music devices independently due to their lack of ability. Reliance on carers was made greater in advanced dementia stages when people with dementia’s capabilities to express their needs were reduced.

The type of arts activity was found to facilitate access and engagement, with music being a particularly accessible form of arts intervention. However, some studies discussed the challenges of tailoring music to people’s personal tastes, particularly in group settings (Inoue et al., 2024; McDermott et al., 2014; Stuart‐Röhm et al., 2024). In Stuart‐Röhm et al., 2024 carers reported that not always knowing songs that were familiar to residents was a barrier to a group singing intervention in care homes and McDermott et al. (2014) highlighted that differences between cultural backgrounds between care home nurses and residents created a disconnect of music taste and less familiarity with residents’ music taste.

Multiple studies based across both residential and community settings highlighted that a lack of staff training was a barrier to engaging with music interventions, particularly in residential settings (Dimopoulos-Bick et al., 2019; Stuart-Rohm et al., 2023; Sung et al., 2011). Sung et al. (2011) explored the use of music in long-term care facilities and the reasons behind its lack of use. This study reported that the majority of participants (69.3%) (nursing staff) did not use music for the residents with dementia in their workplaces with the main reasons being a lack of knowledge, skills, confidence and training to deliver dementia-specific music therapy. This barrier was similarly reported in Stuart-Rohm et al. (2023) with carers expressing that there is a lack of dementia-specific information provided and a need for more dementia-specific training.

In additional to a lack of staff training, a lack of available staff time and commitment to implement and deliver arts interventions was identified as a barrier in multiple studies (Dimopoulos-Bick et al., 2019; Inoue et al., 2024; McDermott et al., 2014; Sung et al., 2011 ; Yous et al., 2024). For example, McDermott et al. (2014) reported that music therapists and staff acknowledged that implementing music therapy in care homes can be challenging due to finding space and time for music sessions within the home’s daily routine. McDermott et al. (2014) highlighted that this required careful planning and negotiation with care home staff which was often challenging due to limited available time and limited interest in music therapy from care staff. Consistent with this, Dimopoulos-Bick et al. (2019) who implemented personalised music playlists into care home settings, found that one of the main barriers to implementation was the time required to establish a personalised playlist for residents creating workflow challenges. Due to the time commitments required to implement the intervention, a key facilitator was a high level of commitment and engagement from staff and in some cases the support of volunteers to reduce demands on staff.

Sustainability of music interventions was a further noted barrier, particularly in community-based settings (Clark et al., 2018; Thompson et al., 2023). Participants in Clark et al.’s (2018) and Thompson et al.’s (2023) study recognised the value of the community singing groups and raised concerns about the ongoing accessibility and future of the group once the research funding ran out.

### Risk of bias

Each of the included studies were screened against the ten questions on the CASP tool (see Table 2). The average CASP score was 9/10 with scores ranging from 8 to 10. Overall the quality ratings were high with one of the most common risks of bias being a lack of consideration of the relationship between researcher and participants. There was a high inter-rater reliability with agreement of ratings reached on 84% of the appraisal items.

**Table 2.** Risk of bias scoring.

Author (year)	Was there a clear statement of the aims of the research?	Is a qualitative methodology appropriate?	Was the research design appropriate to address the aims of the research?	Was the recruitment strategy appropriate to the aims of the research?	Was the data collected in a way that addressed the research issue?	Has the relationship between researcher and participants been adequately considered?	Have ethical issues been taken into consideration?	Was the data analysis sufficiently rigorous?	Is there a clear statement of findings?	How valuable is the research?
Bazooband et al. (2023)	Yes	Yes	Yes	Yes	Yes	Yes	Yes	Yes	Yes	Yes
Clark et al. (2018)	Yes	Yes	Yes	Yes	Yes	No	Yes	Yes	Yes	Yes
Clark et al. (2021)	Yes	Yes	Yes	Yes	Yes	No	Yes	Yes	Yes	Yes
Dahms & Haesner. (2018)	Yes	Yes	Yes	Yes	Yes	No	Yes	Yes	Yes	Yes
Dimopoulos-Bick et al. (2019)	Yes	Yes	Yes	Yes	Yes	No	Yes	No	Yes	Yes
Inoue et al. (2024)	Yes	Yes	Yes	Yes	Yes	No	No	Yes	Yes	Yes
Lee et al. (2022c)	Yes	Yes	Yes	Yes	Yes	Yes	Yes	Yes	Yes	Yes
Lee et al. (2022b)	No	Yes	Yes	Yes	Yes	Yes	Yes	Yes	Yes	Yes
Livingston et al. (2016)	No	Yes	Yes	Yes	Yes	No	Yes	Yes	Yes	Yes
Mangione (2013)	No	Yes	Yes	Yes	Yes	No	Yes	Yes	Yes	Yes
McDermott et al. (2014)	Yes	Yes	Yes	Yes	Yes	No	Yes	Yes	Yes	Yes
Petts and Urmston (2022)	Yes	Yes	Yes	Yes	Yes	No	Yes	Yes	Yes	Yes
Sixsmith et al. (2010)	Yes	Yes	Yes	Yes	Yes	No	Yes	Yes	Yes	Yes
Stuart‐Röhm et al. (2024)	Yes	Yes	Yes	Yes	Yes	Yes	Yes	Yes	Yes	Yes
Stuart-Röhm et al. (2023)	Yes	Yes	Yes	Yes	Yes	No	Yes	Yes	Yes	Yes
Sung et al. (2011)	Yes	Yes	Yes	Yes	Yes	Yes	No	Yes	Yes	Yes
Thompson et al. (2023)	Yes	Yes	Yes	Yes	Yes	Yes	Yes	Yes	Yes	Yes
Unadkat et al. (2017)	Yes	Yes	Yes	Yes	Yes	No	Yes	Yes	Yes	Yes
Yous et al. (2024)	Yes	Yes	Yes	Yes	Yes	No	Yes	Yes	Yes	Yes

## Discussion

This review systematically synthesised existing literature on the barriers and facilitators to accessing and engaging with arts interventions for people with dementia. The 19 studies reviewed encompassed various arts-based interventions, including visual arts and painting, with a majority focusing on music-based programmes. Key barriers identified included limited staff time, insufficient training and commitment, reliance on carers, and concerns about sustainability. Conversely, facilitators included the active involvement of unpaid carers, skilled facilitation, and the adoption of person-centred approaches to enhance engagement and accessibility.

The inclusion of unpaid carers in arts interventions was identified as a key facilitator, aligning with extensive prior research demonstrating the benefits of such interventions for both people with dementia and their carers. Previous studies have shown that arts interventions can enhance quality of life, wellbeing, and the relationship between people with dementia and their carers (Camic et al., 2014; Irons et al., 2020; Schall et al., 2018; Windle et al., 2018). Similarly, this review highlighted that involving carers provided an enjoyable shared activity and offered them respite (Clark et al., 2021; Petts & Urmston, 2022; Thompson et al., 2023). However, despite these benefits, a significant barrier identified was the reliance on unpaid carers to facilitate access to arts interventions. For people with dementia living at home, carers were often essential for enabling participation, whether at home or within community support groups (Dahms & Haesner, 2018; Thompson et al., 2023). This reliance was particularly pronounced in the later stages of dementia, where greater support was required. This finding mirrors broader research on access to services, which has highlighted the disproportionate burden placed on unpaid carers and their critical role in enabling people with dementia to engage effectively with services (Kasper et al., 2015; Quinn et al., 2013). Social services such as day centres, community support programmes, and home care are essential for alleviating the pressures on carers and facilitating access to interventions. A policy implication is the need for greater investment in dementia care services, particularly in supporting unpaid carers. Given the substantial reliance on carers to facilitate access to arts interventions, policymakers should consider more funding for respite care and carer support initiatives to reduce this burden.

Additional support is needed to help people with dementia access these services independently, without over-reliance on carers. For instance, unpaid carers often provide vital assistance with daily activities, appointments, navigating the care system, and accessing community services (Giebel et al., 2023). This role is particularly crucial as individuals with dementia who lack regular carer support are at a disadvantage, often facing poorer health outcomes and reduced access to services (Giebel et al., 2023). In the UK, an estimated 120,000 people with dementia live alone (Dementia UK, 2024), and a lack of regular carer support contributes to creating inequalities. Qualitative research suggests that creating safe and friendly environments, providing enabling rather than disabling care, fostering relationships and community connectedness, and offering appropriate support can help individuals with dementia maintain independence at home (Rapaport et al., 2020). Integrating these factors into arts interventions and community services may reduce reliance on carers while supporting greater independence and may also facilitate access to arts interventions with less reliance on carers. Policymakers should consider integrating arts interventions into existing community support structures, such as dementia-friendly communities and social prescribing initiatives, to reach those without informal carer support.

Workforce facilitation within care settings also emerged as a key factor for enabling access to arts interventions. Studies highlighted that adequate staff time was essential for delivering and facilitating these activities, with insufficient time often acting as a significant barrier (Dimopoulos-Bick et al., 2019; Inoue et al., 2024; Stuart-Röhm et al., 2023; Sung et al., 2011). Several studies noted that support from volunteers helped alleviate some of the pressures faced by care home staff (Dimopoulos-Bick et al., 2019; Mangione, 2013; Yous et al., 2024). However, challenges related to retaining volunteers were also reported (Yous et al., 2024). Policies that provide incentives, such as formal recognition, training opportunities, or stipends, could help attract and retain volunteers. Partnerships between care homes, community organisations, and academic institutions could also be fostered to create sustainable volunteer networks.

Effective staff training was another key consideration, as it promoted confidence and motivation among care staff to deliver arts interventions and engage residents (Dimopoulos-Bick et al., 2019). This aligns with previous research emphasising the importance of training opportunities to equip care home staff with the skills needed to implement participatory arts activities (Dadswell et al., 2020). Additionally, a previous review found that most care staff in long-term dementia care homes experience low to moderate levels of burnout (Costello et al., 2019) highlighting the work demands they face in their existing roles. To address this, integrating arts interventions into existing care practices, as suggested by Dimopoulos-Bick et al. (2019), or prioritising activities that are less demanding on staff, could make implementation more feasible. External facilitation, such as involving arts therapists or volunteers, may reduce the workload on care home staff. However, these approaches can be more costly and potentially less sustainable than in house delivery of arts interventions. Balancing resource demands with the benefits of arts interventions is crucial, as sustainable models of delivery are needed to ensure long-term implementation and effectiveness. To make arts interventions more accessible greater integration of arts interventions into existing dementia care frameworks within care homes and community settings is essential. This could be facilitated by mandated policies or government encouraged training initiatives through accredited bodies, ensuring that arts-based approaches become a standard component of dementia care.

The type of arts intervention was closely linked to its accessibility, with music being highlighted as a particularly engaging and accessible form of intervention. The appropriateness of specific art activities often depends on the stage and progression of dementia. For instance, research indicates that active music interventions are more effective for individuals in the early to moderate stages of dementia, while passive interventions, such as music listening, are better suited for those in advanced stages (Baker et al., 2023). This underscores the importance of selecting arts activities mindfully, tailoring them to the individual’s dementia severity. Different types of interventions may present unique barriers and varying levels of engagement depending on the progression of the condition, emphasising the need for person-centred approaches to maximise accessibility and impact. A common barrier to engagement was the inability to align interventions with participants’ musical tastes in certain situations. These findings underscore the value of integrating person-centred principles when designing and delivering arts interventions. By creating tailored environments and activities that reflect individual preferences and needs, person-centred approaches can significantly enhance access, engagement, and the overall effectiveness of arts interventions for people with dementia.

A person-centred approach is widely recommended for the care of individuals with dementia in both residential and community settings (Edvardsson et al., 2008; Kitwood & Bredin, 1992). This approach emphasises valuing the individual, upholding their personhood, addressing psychological as well as physical needs, and fostering social engagement (Brooker & Latham, 2015; Downs & Lord, 2017). In this review, making mindful accommodations and adaptations to meet individuals’ needs was found to facilitate access to and engagement with arts interventions (Livingston et al., 2016; Mangione, 2013), reinforcing the importance of a person-centred approach in this context. This review also highlighted the importance of tailoring interventions to individuals’ preferences, particularly in music-based programmes.

### Limitations

While this review systematically synthesised evidence and provided an overview of the barriers and facilitators to accessing and engaging with arts interventions for people with dementia, the number of included studies for each type of activity limited our ability to compare these factors across different types of arts interventions, stages of dementia or subtypes of dementia, for example there was only one study that included painting as an activity. It is likely that individuals with dementia experience distinct barriers and facilitators depending on the type of intervention, such as painting versus music, but this could not be assessed in the current review. Furthermore, barriers and facilitators may vary based on dementia severity or dementia subtype and interact with the form of activity; however, this review was unable to determine whether such interactions exist.

Additionally, it should be noted that included studies and parts of the studies that examined barriers and facilitators to engagement were qualitative in nature. Thus, the findings reported in this review reflect participants subjective perceptions, experiences and reported challenges rather than objective measures of accessibility or level of engagement. Qualitative research provides valuable insight into people’s personal experiences however is often shaped by the perspectives of the studies participants and researchers. Due to this, certain barriers and facilitators may be more prominent within the literature because of the themes explored within individual studies and other important barriers and facilitators not identified. Future research would benefit from utilising both qualitative and quantitative methodologies to capture a more holistic understanding of accessibility and engagement with arts interventions across diverse dementia populations.

### Conclusion

This systematic review highlights key facilitators and barriers to accessing and engaging with arts interventions for people with dementia, emphasising the importance of adopting person-centred approaches in their design and delivery. Greater awareness of these factors can inform the development of future interventions which remain necessary to improve the well-being and quality of life of people with dementia. Addressing the barriers to arts interventions for people with dementia requires a multifaceted approach that combines policy reform, workforce development, volunteer engagement, and sustainable funding strategies. By scaling and integrating these solutions into existing care systems, arts interventions can become a more accessible and impactful component of dementia care.

## Supplemental Material

Supplemental Material - Barriers and facilitators to accessing and engaging with arts-based non-pharmacological interventions for people living with dementia: A systematic review Supplemental Material for Barriers and facilitators to accessing and engaging with arts-based non-pharmacological interventions for people living with dementia: A systematic review by Megan Polden, Megan Readman, Tahlia Barnard, Abigail Godfrey and Annabel Gray, and Ashley Sorensen, and Clarissa Giebel in Dementia
