# Supplementary material for: Barriers and facilitators to accessing and engaging with arts-based non-pharmacological interventions for people living with dementia: A systematic review
Source: Dementia (London). 2025 Apr 12;25(4):861–82. doi: 10.1177/14713012251333017 (PMC13061310; doi:10.1177/14713012251333017)
Supplement: Supplemental Material - Barriers and facilitators to accessing and engaging with arts-based non-pharmacological interventions for people living with dementia: A systematic review [file sj-pdf-1-dem-10.1177_14713012251333017.pdf]

Supplementary materials

Full search terms for five databases (PubMed, PsycINFO, CINAHL, Scopus, Web of Science):

((painting OR drawing OR art OR paint* OR draw* OR music* OR sing* OR choir* OR choral* OR performance OR performing OR drama) AND (dementia* OR Alzheimer's Disease* OR neurodegenerative) AND (inequalit* OR inequit* OR access* OR accessible OR accessibility))
